# Anxiety among Pregnant Women Attending Obstetrics Unit of a Tertiary Care Centre during the COVID-19 Pandemic: A Descriptive Cross-sectional Study

**DOI:** 10.31729/jnma.7259

**Published:** 2022-07-31

**Authors:** Surya Prasad Rimal, Kriti Thapa, Ramesh Shrestha

**Affiliations:** 1 Department of Obstetrics and Gynaecology, B.P. Koiraia Institute of Health Sciences, Dharan, Sunsari, Nepal; 2 Department of Psychiatric Nursing, B.P. Koiraia Institute of Health Sciences, Dharan, Sunsari, Nepal

**Keywords:** *anxiety*, *COVID-19*, *pregnancy*

## Abstract

**Introduction:**

The disastrous effect of COVID-19 pandemic on the mental health of vulnerable populations like pregnant women should not be neglected. The objective of the study was to find out the prevalence of anxiety among pregnant women attending the obstetrics unit of a tertiary care centre during the COVID-19 pandemic.

**Methods:**

A descriptive cross-sectional study was conducted from 16 May 2020 to 30 July 2020 among pregnant women attending obstetrics unit of a tertiary care centre. Ethical approval was obtained from the Institutional Review Committee (Reference number: 365/076/077-IRC). Convenience sampling method was used. Pregnancy-related anxiety questionnaires were used and semistructured questionnaires were used for sociodemographic data. Point estimate and 95% Confidence Interval were calculated.

**Results:**

Out of 115 pregnant women, anxiety was found in 21 (18.26%) (11.20-25.32, 95% Confidence Interval).

**Conclusions:**

Anxiety among the pregnant women reported in this study was found to be lower than similar studies conducted in similar settings.

## INTRODUCTION

The potential impact of the COVID-19 pandemic on mental health should not be overlooked, especially in vulnerable populations like pregnant women.^1^ Even under normal circumstances, women are at greater risk of anxiety and depression during pregnancy than at any other time in their lives.^2^ Fear of virus transmission to the foetus, social isolation resulting in reduced support from wider family and friend, disruption of maternal health services etc. make pregnant mothers prone to psychological disruptions.^3^

Previous studies have shown that pregnant women with abnormal levels of anxiety symptoms have doubled during COVID-19.^4,5^ Prenatal mental disorders are associated with miscarriage, preterm birth, low infant birth weight, gestational diabetes and gestational hypertension.^6,7^ The mental health impacts of COVID-19 pandemic have not been extensively assessed in pregnant women, especially in developing countries like Nepal.

The objective of this study was to find out the prevalence of anxiety among pregnant women visiting obstetrics unit of a tertiary care centre during the COVID-19 pandemic.

## METHODS

A descriptive cross-sectional study was conducted from 16 May 2020 to 30 July 2020 among pregnant women attending obstetrics unit of a tertiary care centre during the COVID-19 pandemic. The study was approved by the Institutional Review Committee of the same Institute on 15 May 2020 (Reference number: 365/076/077-IRC). Pregnant women willing to participate during the study period were included in the study. Convenience sampling was done.

The sample size was calculated using the formula:


$$\begin{array}{ll} \text{n} & ={\text{Z}}^{2}\times \frac{\text{p}\times \text{q}}{{\text{e}}^{\text{2}}}\\ & =1.96^{2}\times \frac{0.50\times 0.50}{0.10^{2}}\\ & =97 \end{array}$$

Where,

n = minimum required sample size
Z = 1.96 at 95% Confidence Interval (CI)
p = prevalence taken as 50% for maximum sample size
q = 1-p
e = margin of error, 10%

Taking a 10% non-response rate, the sample size was 107. However, we enrolled 115 participants in this study.

Written informed consent was taken from each participant and confidentiality was maintained. Face to face interview of each participant was conducted in an interview room. The semi-structured questionnaire was used to collect information on socio-demographic profiles. For assessing anxiety pregnancy-related anxiety scale (PrAS) was used. It is a four point likert scale ranging from 1 (never or not at all) to 4 (a lot of the time or very much). The final score on this measure could range from 10 to 40. A total score of 28 and more in nulliparous and 24 or more in multiparous has been taken as the cut off to define pregnancy related anxiety.^8^ PrAS was translated in Nepali and used for data collection.

The data were compiled and analysed in the IBM SPSS Statistics 16.0. Point estimate and 95% CI were calculated.

## RESULTS

Out of 115 pregnant women, pregnancy related anxiety was found in 21 (18.26%) (11.20-25.32, 95% CI). Among them, 3 (14.28%) were primigravida and 18 (85.72%) were multigravida. Mean score of the pregnancy related anxiety score in primigravida and multigravida were 30.33±1.15 and 27.06±4.26 respectively. In this study 15 (71.43%) of the pregnant women consumed alcohol or smoking or both. Among the substance abusers 13 (86.67%) of them consumed alcohol, 1 (6.67%) of them smoked cigarettes and 1 (6.67%) took both the cigarettes and alcohol (Table 1).

**Table 1:** Socio-demographic characteristics of pregnant women with anxiety (n = 21).

Characteristics	Category	n (%)
Age group (years)	>30	15 (71.43)
	>30	6 (28.57)
Religion	Hindu	18 (86.71)
	Buddhist	1 (4.76)
	Muslim	1 (4.76)
	Christian	1 (4.76)
Education	Illiterate	-
	Literate	21 (100)
Level of education	Primary level	4 (19.05)
	Secondary level	12 (57.14)
	Higher education	5 (23.80)
Occupation	Homemaker	13 (61.90)
	Job at private institute	2 (9.52)
	Job at government institute	2 (9.52)
	Own business	2 (9.52)
	Farming	2 (9.52)
Year of marriage (years)	<5	8 (38.09)
	≥5	13 (61.90)
Income	>30,000	8 (38.09)
	30,000-50,000	8 (38.09)
	<50,000	5 (23.81)
Type of family	Nuclear	13 (61.90)
	Joint	8 (38.09)
Residence	Rural	16 (76.20)
	Urban	5 (23.80)
House	Owned	16 (76.20)
	Rented	5 (23.80)

## DISCUSSION

The findings of this study revealed that out of 115 pregnant women, 21 (18.26%) had pregnancy related anxiety. The mental health effect of the COVID-19 pandemic on pregnant women has now become a major public health challenge. This issue is significantly underrepresented and requires appropriate and immediate interventions to avoid adverse health outcomes especially to the vulnerable group like pregnant women.^9^ This is similar to the study done in Iran where PRA was 21%.^10^

Different studies conducted in various parts of the world have reported a wide variety of prevalence of anxiety among pregnant women. Some of the reported prevalence of anxiety are as follows: 39.1% in Nepal,^11^ 11.1% in India,^12^ 36.77% in China,^13^ 37.5% in Nigeria,^14^ 29.7% in Oman,^15^ 29.2% in Turkey,^16^ and 52% in Netherlands.^17^

The prevalence of anxiety reported in this study is lower than most of the other studies conducted in Nepal and other parts of the world. One of the main reasons for this inconsistency might be due to the different instruments used for data collection as these studies have mainly assessed generalised anxiety in pregnant women. The varied distribution of the pandemic might be one of the reasons for the various prevalence rates of the anxiety among various parts of the world.

One of the main reasons for the lower anxiety in this study might be due to the period of the study when the first lockdown was initiated in Nepal. Relatively better COVID related outcomes in Nepal during that period might have also had an influence on the lower rates of anxiety and depression in the pregnant women.^18^

Minimising disruptions of prenatal care perhaps with effective use of telehealth appointments were found not only to reduce vulnerability to stress but also improve resilience among pregnant women.^19^ The telehealth service and the antenatal care services with safety protocols provided by the institute can be another factor for low anxiety and depression in pregnant women.

Though the rates of anxiety in pregnant women were not very high in this study, it still warrants further explore the mental health impact of COVID-19 pandemic on pregnant women. Furthermore, specific interventions need to be formulated to support pregnant individuals during this critical time to mitigate long term negative outcomes.

The study had been conducted only from one hospital. So, the results might not be generalizable to pregnant women of other hospitals. Moreover, in the early days of COVID 19, only a limited number of pregnant women visited the hospital and hence may have limited the findings of this study.

## CONCLUSIONS

The prevalence of pregnancy-related anxiety among the pregnant women during COVID-19 reported in this study was found to be lower than other studies conducted in similar settings. The preventive strategies with strict health protocols complemented by pregnancy counselling services need to be developed and implemented during COVID-19 pandemic.

## References

[1] Dule A (2021). Psychological Distress Among Ethiopian Pregnant Women During COVID-19: Negative Correlation with Self-Efficacy.. Psychol Res Behav Manag..

[2] Priya A, Chaturvedi S, Bhasin SK, Bhatia MS, Radhakrishnan G (2018). Depression, anxiety and stress among pregnant women: a community-based study.. Indian J Psychiatr..

[3] Almeida M, Shrestha AD, Stojanac D, Miller LJ (2020). The impact of the COVID-19 pandemic on women's mental health.. Arch Womens Ment Health..

[4] Lebel C, Kinnon A, Bagshawe M, Tomfohr-Madsen L, Giesbrecht G (2020). Elevated depression and anxiety symptoms among pregnant individuals during the COVID-19 pandemic.. J Affect Disord..

[5] Mappa I, Distefano FA, Rizzo G (2020). Effects of coronavirus 19 pandemic on maternal anxiety during pregnancy: a prospectic observational study.. J Perinat Med..

[6] Qiao Y, Wang J, Li J, Wang J (2012). Effects of depressive and anxiety symptoms during pregnancy on pregnant, obstetric and neonatal outcomes: a follow-up study.. J Obstet Gynaecol..

[7] Field T, Diego M, Hernandez-Reif M, Figueiredo B, Deeds O, Ascencio A (2010). Comorbid depression and anxiety effects on pregnancy and neonatal outcome.. Infant Behav Dev..

[8] Rini CK, Dunkel-Schetter C, Wadhwa PD, Sandman CA (1999). Psychological adaptation and birth outcomes: the role of personal resources, stress, and sociocultural context in pregnancy.. Health Psychol..

[9] Tran BX, Ha GH, Nguyen LH, Vu GT, Hoang MT, Le HT (2020). Studies of Novel Coronavirus Disease 19 (COVID-19) Pandemic: A Global Analysis of Literature.. Int J Environ Res Public Health..

[10] Hamzehgardeshi Z, Omidvar S, Amoli AA, Firouzbakht M (2021). Pregnancy-related anxiety and its associated factors during COVID-19 pandemic in Iranian pregnant women: a web-based cross-sectional study.. BMC Pregnancy and Childbirth..

[11] Moyer CA, Compton SD, Kaselitz E, Muzik M (2020). Pregnancy-related anxiety during COVID-19: a nationwide survey of 2740 pregnant women.. Arch Womens Ment Health..

[12] Tikka SK, Parial S, Pattojoshi A, Bagadia A, Prakash C, Lahiri D (2021). Anxiety among pregnant women during the COVID-19 pandemic in India - A multicentric study.. Asian J Psychiatr..

[13] Shrestha P, Mahato V, Subedi A, Shrestha S (2021). Anxiety and depression in pregnant women amid COVID-19 pandemic.. Asian Journal of Medical Sciences..

[14] Ge Y, Shi C, Wu B, Liu Y, Chen L, Deng Y (2021). Anxiety and adaptation of behavior in pregnant Zhuang women during the COVID-19 pandemic: A Mixed-Mode Survey.. Risk Manag Healthc Policy..

[15] Nwafor JI, Okedo-Alex IN, Ikeotuonye AC (2021). Prevalence and predictors of depression, anxiety, and stress symptoms among pregnant women during COVID-19-related lockdown in Abakaliki, Nigeria.. Malawi Med J..

[16] Sumri ALH, Kindi ALR, Sumri ALS (2021). The psychological effects of the coronavirus Disease 2019 pandemic on pregnant and postpartum women.. J Clin Gynecol Obstet..

[17] Keskin DD, Keskin S, Bostan S (2022). Mental disorders among pregnant women during the COVID-19 pandemic. A cross-sectional study.. Sao Paulo Med J..

[18] Vacaru S, Beijers R, Browne PD, Cloin M, van Bakel H, van den Heuvel MI (2021). The risk and protective factors of heightened prenatal anxiety and depression during the COVID-19 lockdown.. Sci Rep..

[19] Sharma K, Banstola A, Parajuli RR (2021). Assessment of COVID-19 pandemic in Nepal: A lockdown scenario analysis.. Front Public Health.

[20] Preis H, Mahaffey B, Heiselman C, Lobel M (2020). Vulnerability and resilience to pandemic-related stress among U.S. women pregnant at the start of the COVID-19 pandemic.. Soc Sci Med..

